# Epidemiology of *Helicobacter pylori* infection among school-age children (6-15years) in Jalalabad Afghanistan

**DOI:** 10.1186/s12879-025-12041-8

**Published:** 2025-11-26

**Authors:** Abdullah Jan Shinwari, Ahmad Gul Azami

**Affiliations:** 1 Nangarhar Medical Faculty, Nangarhar University, Jalalabad, Afghanistan; 2 Faculty of Tropical Medicine, Mahidol University, Bangkok, Thailand

**Keywords:** *H.* pylori infection, School-aged children, Prevalence, Risk factors, Parental education, Hygiene practices

## Abstract

**Background:**

H. pylori infection poses a significant public health burden in low- and middle-income countries. In children, it can cause gastritis, abdominal pain, anemia, and growth retardation, and increase the risk of peptic ulcers and gastric cancer. This study examines its prevalence and determinants among 6–15-year-old children in Jalalabad, Afghanistan.

**Materials and methods:**

A cross-sectional study was conducted from September 2023 to March 2024 among 460 school-aged children (6–15 years) in Jalalabad, Afghanistan, using multistage stratified random sampling. H. pylori infection was assessed via stool antigen test, and socio-demographic, economic, and hygiene-related factors were analyzed using logistic regression to identify independent risk factors.

**Results:**

Among 460 school-aged children, 49.3% tested positive for H. pylori, with no gender difference. Infection risk was higher in older children (13–15 years) than in those aged 5–9 years (AOR = 1.64, *p* = 0.017), children from larger families (6–12 members: AOR = 2.95, *p* = 0.004; >12 members: AOR = 3.26, *p* = 0.006). Poor hand hygiene before meals ((AOR = 1.73, *p* = 0.012) and after returning home (AOR = 1.85, *p* = 0.007) were independently associated with higher infection risk.

**Conclusion:**

H. pylori infection is common among school-aged children in Jalalabad, linked to older age, large families, low parental literacy, and poor hygiene. Targeted hygiene education and parental awareness programs are needed to prevent infection and long-term gastrointestinal consequences.

**Supplementary Information:**

The online version contains supplementary material available at 10.1186/s12879-025-12041-8.

## Introduction

*Helicobacter pylori* infection is prevalent among children, adolescents and adults globally [1]. Beyond its role in causing chronic gastritis, which can later develop into gastric cancer, it is linked to various extra-gastric conditions, such as iron deficiency anemia and growth retardation in children [2]. Understanding the trends in *H. pylori* infection prevalence among these age groups is crucial for anticipating related diseases, including gastric cancer in adulthood [3, 4].

The prevalence of *H. pylori* infection varies widely based on factors such as age, associated diseases, geographic location, race/ethnicity, socioeconomic status, and hygiene standards [5]. From 1970 to 2015, adult prevalence was reported at 48.5% [6]. A meta-analysis and systematic review in children, covering data until October 2021, found a global prevalence of 32.3% [1]. Between 2000 and 2017, the infection rate was significantly higher in adults (48.6%) than in children (32.6%) [1]. Studies conducted after 2016 indicate a declining prevalence in several countries [7–9]. The latest systematic review and meta-analysis revealed a prevalence of 58.2% between 1980 and 1990, which decreased to 43.1% between 2011 and 2022, showing a marked reduction [8].

Children, unlike adults, typically experience a lower rate of severe disease associated with *H. pylori* infection [9]. However, recent research suggests a potential role of *H. pylori* infection in other digestive diseases, such as gastroesophageal reflux disease (GERD), and several extra-intestinal conditions, including iron deficiency anemia (IDA), growth retardation, idiopathic thrombocytopenic purpura (ITP), asthma, and allergic disorders [10]. Dyspepsia is also prevalent in children with chronic or recurrent abdominal pain (RAP), with up to 80% reporting this symptom [11].

There is a limited number of studies on the epidemiology of *H. pylori* infection and its associated factors among children, where no research has been conducted to date in the Jalalabad city of Afghanistan. Therefore, the aim of this study was to determine the epidemiology and associated factors of *H. pylori* infection in asymptomatic school-aged children in Jalalabad City.

## Materials and methods

### Study design study population, and study area

**Fig. 1:**
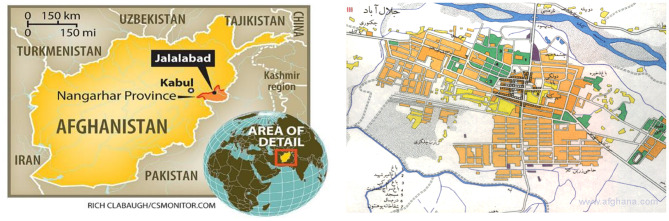
Map of Jalalabad city, Nangarhar, Afghanistan showing the study location

This cross-sectional study was carried out from September 1, 2023 to March 30, 2024, among school-aged students (6–15 years) in Jalalabad city (Fig. 1). Jalalabad is the capital of Nangarhar Province and one of the five major urban centers in Afghanistan. Situated near the Pakistan border, Jalalabad serves as the principal commercial and transit hub of eastern Afghanistan. Located at 34°26′N and 70°26′E, Jalalabad lies in the Kabul River valley at 580 meters’ elevation. The city has a hot semi-arid climate, and its population has grown from 268,637 in 1978 to over one million, concentrated mainly in zones one to five. Covering an area of about 40 square kilometers, Jalalabad contains roughly 40,000 residential units, with 44% of its land designated for agricultural use. Administratively, it comprises eight municipal zones, with the highest population density concentrated in zones one through five.

### Sample size

The sample size was calculated using the single population proportion formula, assuming a 50% prevalence of *H. pylori*, a 95% confidence level (Z = 1.96), and a 5% margin of error (d = 0.05), yielding an initial estimate of 384 participants. To compensate for an anticipated 20% non-response rate, additional participants were recruited, resulting in a total of 460 children enrolled and analyzed.

### Sampling techniques and target population

**Fig. 2:**
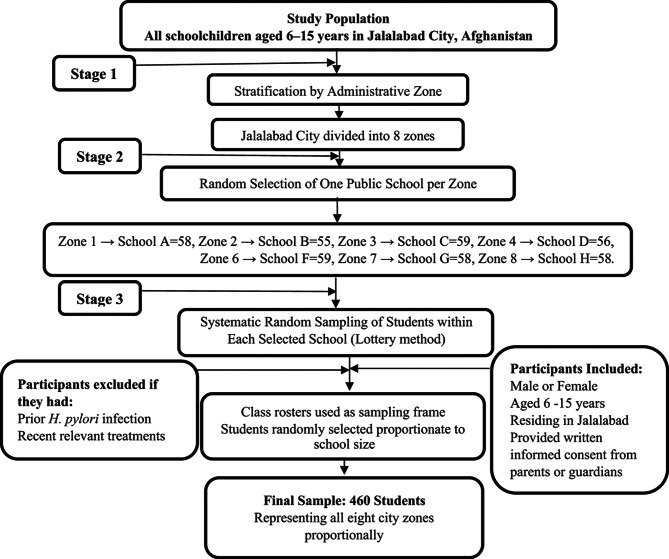
Multistage stratified random sampling process for selecting school-aged participants (6–15 years) in Jalalabad City, Afghanistan

The study population consisted of schoolchildren residing in the eight administrative zones of Jalalabad City. To ensure equitable geographic representation and minimize selection bias, a multistage stratified random sampling approach was employed. In the first stage, Jalalabad City was stratified into its eight administrative zones. In the second stage, one public school from each zone was randomly selected using a simple random (lottery) method (Fig. 2). In the final stage, students were selected from class rosters within each school through systematic random sampling, ensuring proportionate representation by age group and gender. A total of 460 schoolchildren were recruited proportionally across the eight zones of Jalalabad City. The number of participants selected from each school ranged from 55 to 59, based on the total enrollment size of each school (Fig. 2).

### Collection of epidemiological, socio-demographic, and laboratory data on *Helicobacter pylori* infection

A structured questionnaire collected information on age, gender, school, residence, family size, income, parental education, housing type, drinking water source, latrine availability, handwashing practices, handwashing methods, consumption of raw vegetables and street food, fingernail hygiene, and anthelmintic drug use. The inclusion of anthelmintic drug history reflects both exposure to intestinal parasites and general hygiene practices.

The questionnaire was meticulously developed in accordance with previously validated instruments employed in epidemiological investigations of *Helicobacter pylori* and further refined through consultation with experts in gastroenterology, microbiology, and public health. A pretest was conducted among 20 students from a non-participating school to evaluate clarity, linguistic appropriateness, and logical flow. Insights gained from the pilot study informed minor revisions, thereby enhancing comprehension and ensuring content validity. The finalized version was interviewer-administered by trained personnel in classroom settings, using the local language, with individual responses recorded confidentially to minimize peer influence and reporting bias. Each completed questionnaire underwent daily review to ensure completeness and internal consistency. The final instrument is provided in Supplementary File 1.

### Stool sample collection and laboratory analysis

For stool sample collection, selected students or student parents received instructions on hygienic procedures and were provided with labeled, leak-proof containers and plastic sheets to avoid contamination. The following morning, students collected 2–5 g of stool and returned the samples to school, where trained staff transported them to the Microbiology and Parasitology Laboratory at Nangarhar Medical Faculty. Stool samples were tested for *H. pylori* antigens using the One Step RapiCard test strip (Hangzhou Clongene Biotech Co., Ltd, China), a rapid lateral flow chromatographic immunoassay with a reported sensitivity of 94.9%–100% and specificity of 95%–100%. After mixing the sample with diluent, 80 µL was applied to the test cassette. Results were read after 10 min; two lines indicated a positive result.

### Data analysis

Data were analyzed using SPSS version 26. Descriptive statistics were used to summarize variables. Bivariable logistic regression was performed to examine the association between each independent variable and *H. pylori* infection, and variables with *p* < 0.25 were considered candidates for inclusion in the multivariable logistic regression model [12]. Multivariable logistic regression was then conducted to identify independent predictors of *H. pylori* infection, with statistical significance set at *p* < 0.05.

### Data quality management

To ensure data accuracy and reliability, all research staff were trained in standardized data collection procedures before the study began. Questionnaires and case record forms were pretested and refined accordingly. Data collection was supervised daily by the principal investigator, and completed forms were checked for completeness and consistency. Data were double-entered and cross-verified using [SPSS/Excel] to minimize entry errors. Laboratory tests for *H. pylori* detection were performed following manufacturer instructions, with internal quality controls included for each batch.

## Result

A total of 460 school-aged children participated in the study, with a mean age of 11.45 years (SD = 2.50) and a median age of 12 years, ranging from 6 to 15 years. Of these, 227 (49.3%) tested positive and 233 (50.7%) negative for *H. pylori* stool antigen.

### Socio-demographic and household characteristics

The study population comprised 65.4% males and 34.6% females. Most children (75%) lived in households with 6–12 members, and the majority of parents were illiterate (71.5%). Cement-built houses predominated (81.7%), and monthly household income was generally low (< 20,000 AFN) (Table 1).

### Hygiene, lifestyle, and environmental characteristics

Nearly all children (98.9%) had access to flush latrines, while 1.1% used simple latrines. Handwashing practices were suboptimal: 55.7% sometimes washed hands before meals, 16.7% did not wash hands after defecation, and 64% did not wash hands upon returning home. Dietary habits varied, with 50.2% consuming raw vegetables sometimes and 46.6% occasionally eating street food. Drinking water sources included public hand pumps (60%) and bottled water (7.8%). Nail trimming and anthelmintic treatment were inconsistent (Table 1).

### Univariate logistic regression analysis

Univariate analysis (Table 1) showed that children aged 13–15 years had significantly higher odds of *H. pylori* infection compared to those aged 5–9 years (COR: 1.77; 95% CI: 1.23–2.56; *p* = 0.002). Larger household size was strongly associated with infection: 6–12 members (COR: 3.68; 95% CI: 1.93–7.02; *p* < 0.001) and > 12 members (COR: 4.02; 95% CI: 1.88–8.58; *p* < 0.001). Inconsistent handwashing practices, including sometimes washing hands before meals (COR: 1.88; 95% CI: 1.29–2.75; *p* = 0.03) and never washing hands after returning home (COR: 2.08; 95% CI: 1.41–3.07; *p* = 0.02), were associated with higher infection risk. Interestingly, children consuming raw vegetables sometimes had lower odds of infection than those always consuming them (COR: 0.38; 95% CI: 0.18–0.83; *p* = 0.043). Other factors, including gender, parental education, house type, toilet type, monthly income, street food consumption, handwashing method, vegetable washing, drinking water source, nail trimming, and anthelmintic use, were not significantly associated with infection.

**Table 1:** Socio-demographic, hygiene, environmental, lifestyle, and health-related characteristics of school-aged children in Jalalabad, Afghanistan (*n* = 460), and univariate logistic regression analysis of Helicobacter pylori infection prevalence

Variable	Stool H. pylori Ag Result		Total N (*n* = 460)	Univariate logistic regression	
	Positive *n*(%)	Negative *n*(%)		COR (95% CI)	*p* value
**Gender**					.
Male	150(49.8%)	151(5.2%)	301	1 (reference)	-
Female	77(48.4%)	82(51.6%)	159	0.93 (0.65–1.33)	0.71
**Age**					
5–9	56(47.5%)	62(52.5%)	118	1 (reference)	-
10–12	65(52.8%)	58(47.2%)	123	1.28 (0.87–1.88)	0.20
13–15	121(55.3%)	98(44.7%)	219	1.77 (1.23–2.56)	0.002
**Numbers of family members**					
< 6	20(37.7%)	33(62.3%)	53	1 (reference)	-
6–12	202(58.5%)	143(41.5)	345	3.68 (1.93–7.02)	< 0.001
> 12	40(64.5%)	22(35.5%)	62	4.02 (1.88–8.58)	< 0.001
**Parent Educational Status**					.
Illiterate	203(61.7%)	126(38.3%)	329	0.894(0.418–1.912)	0.774
Primary	19(45.2%)	23(54.8%)	42	1.130(0.438–2.917)	0.801
Secondary	14(43.8%)	18(56.3%)	32	1.200(0.437–3.292)	0.723
Higher	10(35.7%)	18(64.3%)	28	1.244(0.438–3.536)	0.682
University	10(34.5%)	19(65.5%)	29	1 (reference)	
**House type**					
Cement House	186(49.5%)	190(50.5%)	376	1 (reference)	-
Mud made House	41(48.8%)	43(51.2%)	84	0.97 (0.58–1.62)	0.92
**Type of Toilet**					
Sample Latrine	3(60%)	2(40%)	5	1.467(0.243–8.865)	0.676
Flash Latrine	225(56%)	230(44%)	455	1 (reference)	-
**Monthly income (Afg) of the family**					
< 10,000	168(62%)	103(38%)	271	1.808(0.424–7.715)	0.424
10,000–20,000	85(53.9%)	70(46.1%)	152	1.901(0.439–8.240)	0.390
20,000–30,000	12(41.4%)	17(58.6%)	29	0.635(0.122–3.295)	0.589
High > 30,000	3(37.5%)	8(62.5%)	8	1(reference)	-
**Eating raw vegetable**				.	
Always	37(72.5%)	11(27.5%)	51	Reference	
Sometime	207(50.9%)	200(49.1%)	407	0.38(0.18–0.83)	0. 0429
Never	1(50%)	1(50%)	2	0.30 (0.01–7.63)	0. 801
**Eating street food**					
Always	23(46%)	27(54%)	50	Reference	
Sometime	160(46.6%)	143(53.4%)	303	1.02 (0.55–1.87)	0.132
Never	44(41.2%)	6358.8(%)	107	0.82 (0.43–1.56)	0.941
**Hand washing before meal**					
Always	107(41.8%)	149(58.2%)	256	Reference	
Sometime	118(57.8%)	86(42.8%)	204	1.88 (1.29–2.75)	0.03
**Hand washing after defecation**					
Always	144(37.6%)	239(62.4%)	383	Reference	
Sometime	41(53.2%)	36(46.8%)	77	1.88 (1.13–3.13)	0. 731
**Washing hands back home**					
Sometime	87(52.4%)	79(47.6%)	166	Reference	
Never	198(67.3%)	96(32.7%)	294	2.08 (1.41–3.07)	0.02
**Type of hand washing**					
Wipe with cloth	1	0	1		
Only water	133(51.8%)	124(48.2%)	257	1.116(0.772–1.613)	0.443
Soap	99(49%)	103(51%)	202	Reference	
**Washing vegetable before eating**					
Always	176(41.2%)	251(58.8%)	427	Reference	
Sometime	19(57.6%)	14(42.4%)	33	1.94 (0.94–3.99)	0.689
**Drinking water source**					
PW + PTW	29(58%)	21(42%)	50	1.014(0.425–2.416)	0.805
PTW	5(71.4%)	2(28.6%)	7	0.560(0.096–3.283)	0.532
PHP	126(45.6%)	150(54.3%)	276	1.667(0.825–3.369)	0.294
Neb	6(60%)	4(40%)	10	0.933(0.224–3.893)	0.888
Wd	38(46.9%)	43(53.1%)	81	1.435(0.649–3.171)	0.551
Bottle	15(41.7%)	21(58.3%)	36	Reference	
**Nail cutting time**					
Once a week	188(46.7%)	215(53.7%)	403	Reference	
Once in 2weeks	30(53.6%)	26(46.4%)	56	1.30 (0.74–2.30)	0.712
Once in 3weeks	1(100%)		1		
**Taken anthelmintic drugs**					
Never	4(40%)	6(60%)	10	1.453(0.397–5.315)	0.396
Less than six months	130(49.8%)	131(50.2%)	261	0.976(0.671–1.420)	0.489
More than six months	93(49.2%)	96(50.8%)	189	Reference	

Note: AFN = Afghan Afghani, PW + PTW = Pipe Water and Private Tube Well; PTW = Private Tube Well; PHP = Public Hand Pump; Neb = Neighbour; Wd = Water Dispenser

### Multivariable logistic regression analysis

Variables with *p* < 0.25 in univariate analysis were included in the multivariable model to control for confounding (Table 2). Children aged 13–15 years remained at higher risk (AOR: 1.64; 95% CI: 1.09–2.47; *p* = 0.017). Larger household size was independently associated with infection: 6–12 members (AOR: 2.95; 95% CI: 1.42–6.10; *p* = 0.004) and > 12 members (AOR: 3.26; 95% CI: 1.41–7.55; *p* = 0.006). Suboptimal handwashing—sometimes before meals (AOR: 1.73; 95% CI: 1.13–2.64; *p* = 0.012) and never after returning home (AOR: 1.85; 95% CI: 1.18–2.89; *p* = 0.007)—was also independently associated with higher odds of infection, emphasizing the role of hygiene behaviors in *H. pylori* transmission.

**Table 2:** Univariable and multivariable logistic regression analyses of potential risk factors associated with H. pylori infection in school age student in Jalalabad city, Afghanistan (*N* = 460)

Variable	Crude OR (95%, CI)	*p*-value	Adjusted OR (95%, CI)	*p*-value
Age group (13–15 years)	1.77 (1.23–2.56)	0.002	1.64 (1.09–2.47)	0.017
Numbers of family members (6–12)	3.68 (1.93–7.02)	< 0.001	2.95 (1.42–6.10)	0.004
Numbers of family members (> 12)	4.02 (1.88–8.58)	< 0.001	3.26 (1.41–7.55)	0.006
Only sometimes handwashing before meal	1.88 (1.29–2.75)	0.001	1.73 (1.13–2.64)	0.012
Never washing after returning home	2.08 (1.41–3.07)	0.001	1.85 (1.18–2.89)	0.007

## Discussion

This study found a high prevalence of *H. pylori* infection (49.3%) among asymptomatic school-aged children in Jalalabad, Afghanistan, **which is comparable to the 45.1% reported in Menoufia**,** Egypt** [13]. Even higher prevalence rates have been observed in other regions, including Egypt (72.38%) [14], Pakistan (72.3%) [15], and Vietnam (87.7%) [16]. In contrast to these regional findings, global estimates from systematic reviews conducted by Yuan C et al. [1] and Zamani M et al. [7] report lower average prevalence rates of 32.4% and 32.6%, respectively, indicating that the burden of infection in Jalalabad exceeds the global average. Much lower prevalence rates have also been reported in Sub-Saharan Africa (14.2%), Poland (23.6%) [18, 19], and Sudan (8.4%) [20] compared to the 49.3% observed in the present study. These contrasts highlight the influence of regional, socioeconomic, and environmental factors on *H. pylori* infection patterns.

In this study, no significant difference was found in *H. pylori* prevalence between males (49.8%) and females (48.4%), consistent with prior research indicating infection is influenced more by environmental and socioeconomic factors than gender [1, 7, 17, 20]. In the present study, *H. pylori* prevalence increased with age, from 47.5% in children aged 5–9 years to 55.3% in those aged 13–15 years, indicating higher infection risk among older school-aged children. This trend aligns with Yuan C et al.’s systematic review, which reported a progressive increase in infection rates from early childhood through adolescence [22]. Similarly, in Vietnam, Che TH, Nguyen Cam T, Ngo DTT, et al. (2022) observed an overall prevalence of 87.7% among school-aged children (6–15 years) in Ho Chi Minh City, with the highest infection rate recorded in children aged 9–11 years and a slight decline in older adolescents [16, 23]. These findings underscore the age-related dynamics of *H. pylori* infection and suggest that older school-aged children may warrant particular attention in surveillance and prevention efforts.

The current study identified key factors influencing *H. pylori* infection in children, including large family size, low parental education, and low family income. Occasional consumption of raw vegetables or fruits was linked to lower infection risk, suggesting dietary habits related to food hygiene affect transmission. Poor hygiene, especially inconsistent handwashing, was strongly associated with higher infection rates, highlighting the fecal-oral transmission route. These findings consistence with previous research: Sally A et al., 2021 found poor hygiene, municipal tap water use, and contact with domestic animals increased infection risk [13], while Che TH et al., 2023 reported infrequent handwashing, crowded living, and large families as risk factors in Vietnam [16]. Yuan C et al., 2022’s systematic review (2022) similarly emphasized low economic status, multiple siblings, poor sanitation, and parental education as contributors [1]. Other studies by Hooi JK et al., 2017 and Mohammad MA et al., 2008 also confirm socio-economic and environmental determinants as critical in *H. pylori* infection [6, 14]. These findings highlight that both personal hygiene and broader socio-environmental factors play critical roles in *H. pylori* transmission across various populations. These findings highlight that both personal hygiene and broader socio-environmental factors play critical roles in *H. pylori* transmission across various populations.

The present study found a high prevalence of *H. pylori* infection (49.3%) among school-aged children in Jalalabad, indicating a substantial public health burden. Given that *H. pylori* is a major cause of chronic gastritis, peptic ulcer disease, and a recognized risk factor for gastric adenocarcinoma and MALT lymphoma [23], these findings highlight potential long-term gastrointestinal risks. Chronic infection may also contribute to extra-gastric manifestations, including iron deficiency anemia, vitamin B12 deficiency, and functional gastrointestinal disorders such as dyspepsia and irritable bowel syndrome, which can impair growth and quality of life [24–26]. In high-prevalence settings, untreated childhood infection may further predispose to metabolic and cardiovascular complications later in life, emphasizing the importance of early detection, health education, and eradication strategies to mitigate both immediate and long-term consequences.

Given the high prevalence of *H. pylori* infection (49.3%) observed in this study, early detection and risk stratification are critical to prevent long-term gastrointestinal and extra-gastric complications. Emerging machine learning and AI–based approaches have shown promise in gastrointestinal disorders, including functional dyspepsia and gastric cancer, by identifying disease subgroups and predicting prognosis [27–29]. In high-prevalence populations, such as Afghan school-aged children, these tools could complement conventional diagnostic strategies, enabling timely intervention and targeted prevention programs.

## Strengths and limitations

This study provides valuable baseline data on *H. pylori* infection among schoolchildren in Jalalabad, Afghanistan. Strengths include a large, randomly selected sample, use of a validated stool antigen test, and robust multivariate analysis based on a pretested questionnaire. However, as a cross-sectional study, it cannot determine causality. Its findings may not be generalizable beyond school-attending children in one city. Possible recall bias and reliance solely on stool antigen testing without confirmatory diagnostics also limit accuracy.

## Conclusion

This study demonstrated a high prevalence of *Helicobacter pylori* infection among school-aged children in Jalalabad, Afghanistan, emphasizing that the infection remains a significant public health concern in this setting. Age-related increase in infection rates highlights the cumulative risk of exposure during childhood, while hygiene, environmental, and socioeconomic conditions continue to play critical roles in transmission dynamics. These findings underscore the need for integrated, school- and community-based prevention strategies focusing on health education, improved sanitation, and safe water access. Strengthening diagnostic capacity through affordable stool antigen testing can enhance early detection and management, particularly in resource-limited settings. Moreover, future research should employ standardized sampling and advanced analytical approaches, such as multivariable regression and AI-assisted models, to further elucidate transmission pathways and guide evidence-based interventions. Collectively, addressing *H. pylori* infection in childhood could substantially reduce the long-term burden of gastrointestinal and extra-gastric diseases in Afghanistan and similar endemic regions.

## Supplementary Information

Below is the link to the electronic supplementary material.

Supplementary Material 1

## Data Availability

The datasets used and/or analyzed during the current study are available from the corresponding author on reasonable request.

## References

[1] Yuan C, Adeloye D, Luk TT, Huang L, He Y, Xu Y, et al. The global prevalence of and factors associated with Helicobacter pylori infection in children: a systematic review and meta-analysis. Lancet Child Adolesc Health. 2022;6(3):185–94. 35085494 10.1016/S2352-4642(21)00400-4

[2] Hamdan SZ, Hamdan HZ, Nimieri M, Adam I. The association betweenHelicobacter pylori Infection and iron Deficiency anemia in children: asystematic review and meta-analysis. Journal of Pediatric Infectious Diseases.2022;17(02):59–70. 10.1055/s-0042-1743502

[3] Borka Balas R, Meliț LE, Mărginean CO. Worldwide prevalence and risk factors of Helicobacter pylori infection in children. Children. 2022;9(9):1359. 10.3390/children9091359 10.3390/children9091359 PMC9498111 36138669

[4] Park JS, Jun JS, Seo J-H, Youn H-S, Rhee K-H. Changing prevalence of Helicobacter pylori infection in children and adolescents. Clin Experimental Pediatr. 2021;64(1):21. 10.3345/cep.2019.01543 10.3345/cep.2019.01543 PMC7806412 32668822

[5] Malfertheiner P, Camargo MC, El-Omar E, Liou J-M, Peek R, Schulz C, et al. Helicobacter pylori infection. Nat Reviews Disease Primers. 2023;9(1):19. 37081005 10.1038/s41572-023-00431-8 PMC11558793

[6] Hooi JK, Lai WY, Ng WK, Suen MM, Underwood FE, Tanyingoh D, et al. Global prevalence of Helicobacter pylori infection: systematic review and meta-analysis. Gastroenterology. 2017;153(2):420–9. 28456631 10.1053/j.gastro.2017.04.022

[7] Zamani M, Ebrahimtabar F, Zamani V, Miller W, Alizadeh‐Navaei R, Shokri‐Shirvani J, et al. Systematic review with meta‐analysis: the worldwide prevalence of Helicobacter pylori infection. Aliment Pharmacol Ther. 2018;47(7):868–76. 10.1111/apt.14561 29430669

[8] Li Y, Choi H, Leung K, Jiang F, Graham DY, Leung WK. Global prevalence of Helicobacter pylori infection between 1980 and 2022: a systematic review and meta-analysis. Lancet Gastroenterol Hepatol. 2023;8(6):553–64. 37086739 10.1016/S2468-1253(23)00070-5

[9] Ravikumara M. Helicobacter pylori in children: think before you kill the bug! Therapeutic Adv Gastroenterol. 2023;16:17562848231177610. 10.1177/17562848231177610 PMC10285598 37361453

[10] Pellicano R, Franceschi F, Saracco G, Fagoonee S, Roccarina D, Gasbarrini A. Helicobacters and extragastric diseases. Helicobacter. 2009;14:58–68. 19712170 10.1111/j.1523-5378.2009.00699.x

[11] Perez ME, Youssef NN. Dyspepsia in childhood and adolescence: insights and treatment considerations. Curr Gastroenterol Rep. 2007;9(6):447–55. 18377794 10.1007/s11894-007-0058-4

[12] Bursac Z, Gauss CH, Williams DK, Hosmer DW. Purposeful selection of variables in logistic regression. Source Code Biol Med. 2008;3:1–8. 19087314 10.1186/1751-0473-3-17 PMC2633005

[13] El Shazli HA, Al Bahnasy RE, Badr SA, Mehesin SA, Soliman SS, Ghoneim YA. Epidemiology of Helicobacter pylori infection among children (6–12 years) in menoufia Governorate. Menoufia Med J. 2021;34(1):354–9.

[14] Mohammad MA, Hussein L, Coward A, Jackson SJ. Prevalence of Helicobacter pylori infection among Egyptian children: impact of social background and effect on growth. Public Health Nutr. 2008;11(3):230–6. 17666124 10.1017/S1368980007000481

[15] Ahmad T, Bilal R, Khanum A. Prevalence of Helicobacter pylori infection in school going children of Bhara Kahu area, Islamabad. Pakistan Institute of Nuclear Science and Technology; 2009.

[16] Che TH, Nguyen TC, Vu VNT, Nguyen HT, Hoang DTP, Ngo XM, et al. Factors associated with Helicobacter pylori infection among school-aged children from a high prevalence area in Vietnam. Int J Public Health. 2023;68:1605908. 37251301 10.3389/ijph.2023.1605908 PMC10209423

[17] Ahmad T, Bilal R, Khanum A. Prevalence of helicobacter pylori infection in school going children of Bhara Kahu area, Islamabad. Pakistan Institute of Nuclear Science and Technology; 2009.

[18] Awuku YA, Simpong DL, Alhassan IK, Tuoyire DA, Afaa T, Adu P. Prevalence of Helicobacter pylori infection among children living in a rural setting in Sub-Saharan Africa. BMC Public Health. 2017;17:1–6. 28438158 10.1186/s12889-017-4274-z PMC5404296

[19] Szaflarska-Popławska A, Soroczyńska-Wrzyszcz A. Effect of Helicobacter pylori infection on nutritional status in Polish teenagers. Gastroenterol Res Pract. 2021;2021(1):6678687. 33859683 10.1155/2021/6678687 PMC8024096

[20] Alshareef SA, Hassan AA, Abdelrahman DN, AlEed A, Al-Nafeesah A, Adam I. The prevalence and associated factors of Helicobacter pylori infection among asymptomatic adolescent schoolchildren in sudan: a cross-sectional study. BMC Pediatr. 2023;23(1):582. 37985974 10.1186/s12887-023-04411-5 PMC10662923

[21] Alshareef SA, Hassan AA, Abdelrahman DN, AlEed A, Al-Nafeesah A, Adam I. The prevalence and associated factors of Helicobacter pylori infection among asymptomatic adolescent schoolchildren in Sudan: a cross-sectional study. BMC Pediatr. 2023;23(1):582. 10.1186/s12887-023-04411-5 PMC10662923 37985974

[22] Che TH, Nguyen TC, Ngo DTT, Nguyen HT, Vo KT, Ngo XM, et al. High prevalence of Helicobacter pylori infection among school-aged children in Ho Chi Minh City, Vietnam. Int J Public Health. 2022;67:1605354. 36439280 10.3389/ijph.2022.1605354 PMC9684168

[23] Khazaaleh S, Alomari M, Rashid MU, Castaneda D, Castro FJ. Gastric intestinal metaplasia and gastric cancer prevention: watchful waiting. Cleve Clin J Med. 2024;91(1):33–9. 38167394 10.3949/ccjm.91a.23015

[24] Tsay F-W, Hsu P-I. H. pylori infection and extra-gastroduodenal diseases. J Biomed Sci. 2018;25(1):65. 30157866 10.1186/s12929-018-0469-6 PMC6114542

[25] Wang Z, Tan W, Xiong H, Huang J, Wei H, Li M, et al. Impact of Helicobacter pylori infection on iron deficiency anemia in children: a systematic review and meta-analysis with early intervention implications. Front Microbiol. 2025;16:1541011. 40611956 10.3389/fmicb.2025.1541011 PMC12222266

[26] Black CJ, Drossman DA, Talley NJ, Ruddy J, Ford AC. Functional Gastrointestinal disorders: advances in Understanding and management. Lancet. 2020;396(10263):1664–74. 33049221 10.1016/S0140-6736(20)32115-2

[27] Liu Y, Liu Y, Ye S, Feng H, Ma L. A new ferroptosis-related signature model including messenger RNAs and long non-coding RNAs predicts the prognosis of gastric cancer patients. J Translational Intern Med. 2023;11(2):145–55. 10.2478/jtim-2023-0089 PMC10680379 38025952

[28] Zha Y, Xue C, Liu Y, Ni J, De La Fuente JM, Cui D. Artificial intelligence in theranostics of gastric cancer, a review. Med Rev. 2023;3(3):214–29. 10.1515/mr-2022-0042 PMC10542883 37789960

[29] Zhu L, Xu S, Guo H, Lu S, Gao J, Hu N, et al. Machine learning-based phenogroups and prediction model in patients with functional Gastrointestinal disorders to reveal distinct disease subsets associated with gas production. J Translational Intern Med. 2024;12(4):355–66. 10.2478/jtim-2024-0009 PMC11444472 39360163

