# Supplementary material for: Epidemiology of Helicobacter pylori infection among school-age children (6-15years) in Jalalabad Afghanistan
Source: BMC Infect Dis. 2025 Nov 26;25:1659. doi: 10.1186/s12879-025-12041-8 (PMC12659387; doi:10.1186/s12879-025-12041-8)
Supplement: Supplementary file 1 — Supplementary Material 1 [file 12879_2025_12041_MOESM1_ESM.pdf]

QUESTIONNAIRE FOR THE EPIDEMIOLOGY OF HELICOBACTER PYLORI INFECTION AMONG SCHOOL GOING CHILDREN (6-15YEARS) IN JALALABAD AFGHANISTAN

Basic information

Site : ☐☐

Student ID : ☐☐☐

Date of visit : ☐☐ / ☐☐ / ☐☐☐☐

dd / mm / yyyy

Demographic information

Age: ☐ ☐ years

Sex : ☐ 1. male ☐ 2. Female

Residence : ☐ 1. Urban ☐ 2 . rural

Family size and education

1-Number of family members : ☐ 1. Less than 6 ☐ 2. 6 to 12

☐ 3. More than 12

2-Parents education level : ☐ 1. Did not study ☐ 2. Elementary school

☐ 3. high school ☐ 4.high vocational diploma

☐ 5. Bachelor and high

3-Family income per month : ☐ 1 . less than 10000 afs ☐ 2. 10000 to 20000 afs

☐ 3. 20000 to 30000 afs ☐ 4 . more than 30000 afs

Characteristics of house

4-Type of house : ☐ 1 . cement house ☐ 2. Mud made

5-Does your house have toilet? ☐ 1. no ☐ 2.yes specify

6-Types of toilets : ☐ 1. Flash ☐ 2.latrin

☐ 3. Open area ☐ 4 . farms

Habits

7-Does your child eat raw vegetable and fruits?

☐1. Always ☐2. Sometimes ☐3. No

8-Does the child eats raw meat? ☐1.yes ☐2 . No

9-Does the child eat street food?

☐1. Always ☐2. Sometimes ☐3. No

Personal hygiene

10-Does the child wash his/her hands before meal? (you can select only one)

☐1. Always ☐2 . Sometime ☐3. Never

11-Does the child was his/her hands after defection? (you can select only one).

☐1. Always ☐2 . Sometime ☐3. Never

12-Does the child wash his/her hands in any of the following ways?

☐1. Wipe with clean cloth ☐2 .only water ☐3.water and soap

13-Does the child wash fresh vegetable before eating?

☐1. Always ☐2. Sometimes ☐3. No

14-Where does the child use drinking water from?

☐1. PW ☐2. PW + PTW ☐3 . PTW ☐4. PHP ☐5. Neb ☐6.WD ☐7.Bottle

15-Finger nail hygiene: (nail cutting time)

☐1. once a week ☐2. Once in two week ☐3.once in three week

Result of Lab Investigation

16-Result of *H. Pylori* Stool Antigen Test

☐1. Positive ☐2. Negative

PW (People utilizing pipe water), PTW (Private tube well, PHP (public hand pump), NEB (utilizing water from neighbor
